# Primary mediastinal choriocarcinoma requiring differentiation from non-small cell lung cancer: An autopsy case report

**DOI:** 10.1016/j.rmcr.2024.102037

**Published:** 2024-05-15

**Authors:** Takahiro Matsuyama, Koji Kubota, Kentaro Tsuruzono, Hiroko Uchida, Tetsuro Hamasaki, Keiko Mizuno, Hiromasa Inoue

**Affiliations:** a Department of Pulmonary Medicine, Imakiire General Hospital, 43-25 Kouraicho, Kagoshima City, 890-0051, Kagoshima, Japan; b Department of Pulmonary Medicine, Graduate School of Medical and Dental Sciences, Kagoshima University, 8-35-1 Sakuragaoka, Kagoshima City, 890-8520, Kagoshima, Japan

**Keywords:** Autopsy, Choriocarcinoma, Mediastinal tumor, Serum β-human chorionic gonadotropin

## Abstract

A 65-year-old man with dyspnea and hemoptysis presented with a right upper lobe mass associated with enlarged mediastinal lymph nodes and bilateral pulmonary nodules on chest computed tomography (CT), suspected lung cancer. Bronchial and CT-guided biopsies revealed poorly differentiated carcinoma. His condition deteriorated rapidly before a definitive diagnosis could be made. Autopsy revealed primary mediastinal choriocarcinoma. Primary mediastinal choriocarcinomas are rare, difficult to diagnose early and have a poor prognosis. In patients with a tumor expanding across the lung and mediastinum and exhibiting pathologic findings of a pooly differentiated carcinoma, we should consider choriocarcinoma, evaluating the serum β-human chorionic gonadotropin levels.

## 1 Introduction

Primary mediastinal choriocarcinomas are rare and most frequently occur in young patients [1]. In most cases, the disease has progressed when symptoms appear. As choriocarcinomas grow rapidly, early metastasis is frequently observed and the prognosis is poor [1,2]. Consequently, making an early diagnosis is difficult for clinicians and pathologists. Herein, we report the case of an older patient with primary mediastinal choriocarcinoma that was not definitively diagnosed before death, identified at autopsy.

## 2 Case presentation

**Fig. 1:**
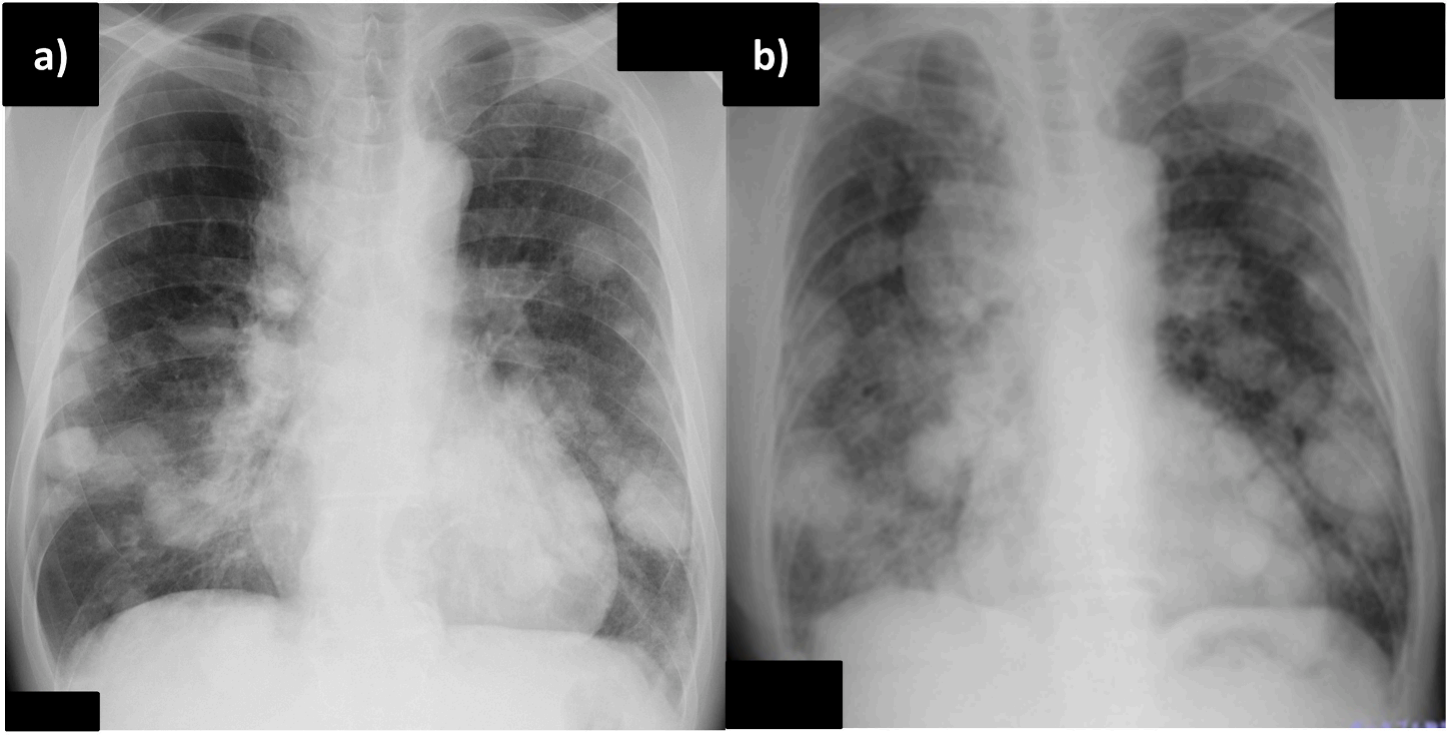
Chest radiograph showing multiple nodules and tumors in the bilateral lung fields a) on admission and b) on the 20th day of hospitalization.

**Fig. 2:**
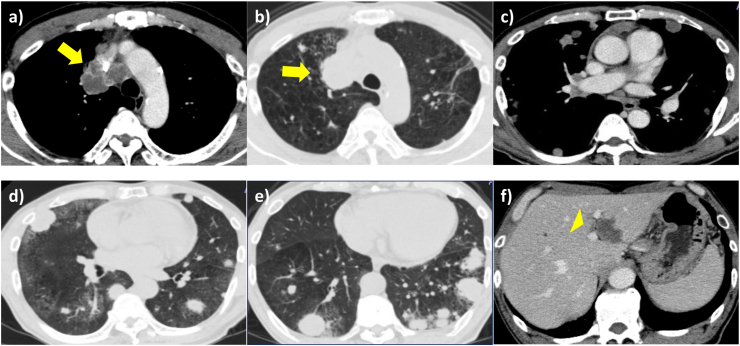
Chest computed tomography obtained on admission showing a-e) a right upper lobe tumor with enlarged mediastinal lymph nodes (arrow), tumors in the bilateral lung fields and f) a low-density area within the liver (arrowhead).

A 65-year-old man was referred to our hospital because of hemoptysis and dyspnea. Physical examination revealed mild facial edema and finger clubbing, but no abnormal breath sounds were heard on chest auscultation. A chest radiograph showed an enlarged superior mediastinum and multiple nodules in the bilateral lung fields (Fig. 1a). Chest computed tomography (CT) revealed multiple nodules in the bilateral lung fields, compression of the superior vena cava by a right upper lobe mass and enlarged mediastinal lymph nodes, enlarged right hilar lymph nodes, and a low-density area within the liver (Fig. 2). Diffusion-weighted magnetic resonance imaging showed a high-intensity lesion in the left occipital lobe, indicative of tumor embolization, but no contrast effect was observed. Laboratory examination revealed elevated levels of transaminase, C-reactive protein, carcinoembryonic antigen (7.1 ng/ml), and cytokeratin 19 fragment antigen (29.7 ng/ml). Sputum cytological examination revealed findings that raised a suspicion of adenocarcinoma. However, both of a bronchoscopic and CT-guided biopsies of the right lower lung mass revealed poorly differentiated carcinoma. On immunostaining, the specimen was positive only for anti-cytokeratin 5.2. It was negative for thyroid transcription factor 1 (TTF-1), napsin, cytokeratin 5/6, synaptophysin and chromogranin A. Based on the imaging and pathological findings, the patient was diagnosed with cT4N2M1c stage IVB non-small cell lung cancer with superior vena cava syndrome, and treatment with systemic chemotherapy was planned. However, his respiratory condition and performance status worsened rapidly, with rapid enlargement of the bilateral lung tumors noted on a chest radiograph (Fig. 1b). He was treated with oxygen and steroids for symptom alleviation and died on the 31st day of admission.

**Fig. 3:**
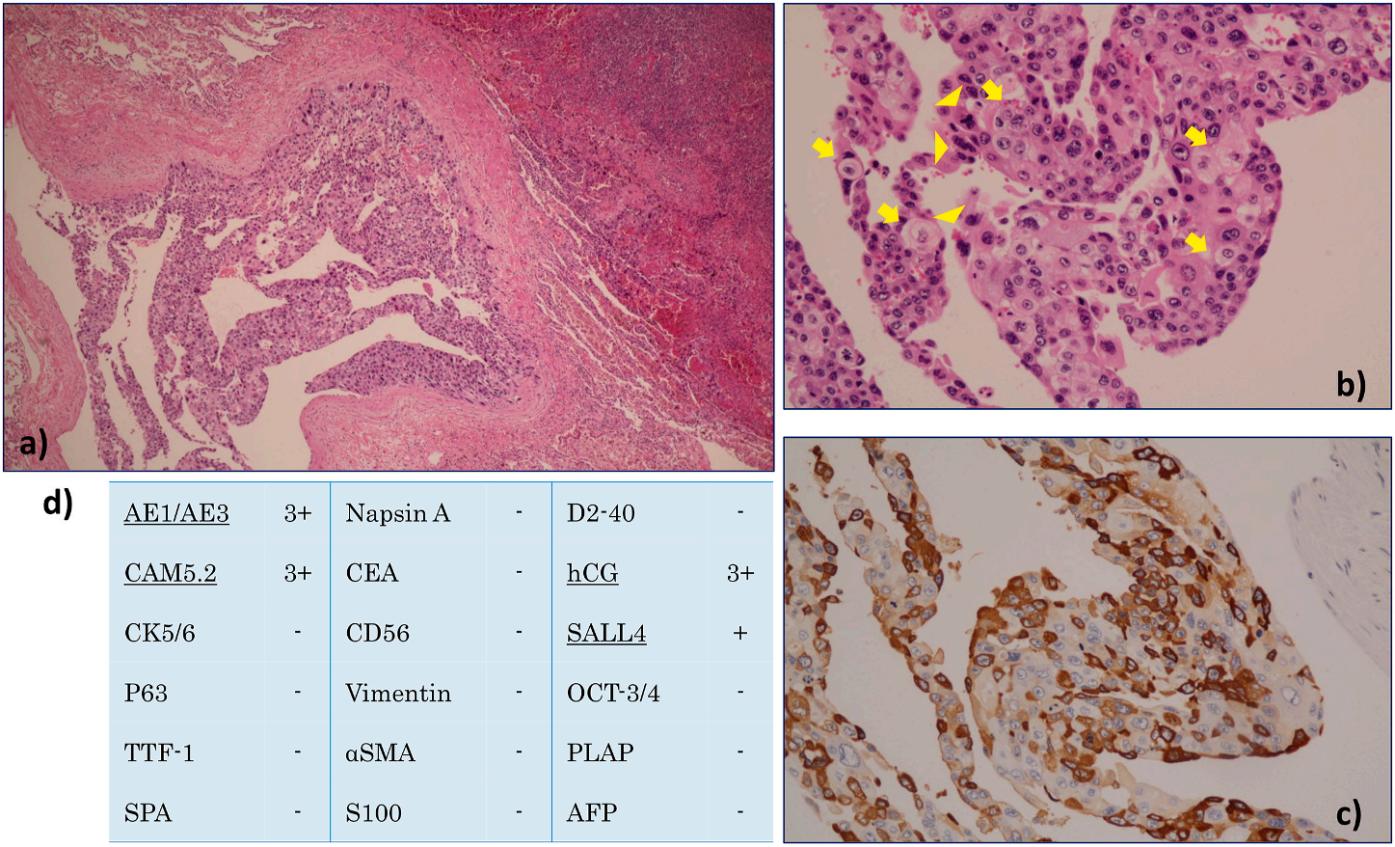
Histological findings. An autopsy specimen of a right upper lobe tumor stained with hematoxylin and eosin shows a, b) cytotrophoblasts (arrow) and syncytiotrophoblasts (arrowhead) stained with hematoxylin and eosin. c) On immunostaining, tumor cells show positivity for human chorionic gonadotropin (hCG). d) Positive immunostaining results for cytokeratin AE1/AE3, anti-cytokeratin 5.2 (CAM5.2), sal-like protein 4 (SALL4) and hCG.

Autopsy revealed a large tumor extending across the right upper lobe and mediastinum and numerous bilateral lung nodules. Most parts of the tumor were reddish, indicative of hemorrhage, with some white areas. The tumor was primarily located in the mediastinum and appeared to compress the right upper lobe. No abnormal findings were observed in the testes or other organs thought to be the primary site. Consequently, the mediastinum was considered the primary site. Histological examination of the mediastinal tumor revealed polymorphous tumor cells with necrosis, indicative of cytotrophoblasts and syncytiotrophoblasts (Fig. 3a and b). Immunostaining revealed that the tumor cells were positive for human chorionic gonadotropin (hCG), sal-like protein 4 and cytokeratin, and negative for lung adenocarcinoma markers such as TTF-1, surfactant protein A and napsin (Fig. 3c and d). Metastatic lesions were observed in the brain, stomach, jejunum, liver, right kidney, left adrenal gland, sigmoid colon, and abdominal wall. Based on these findings, a final diagnosis of primary mediastinal choriocarcinoma was made.

## 3 Discussion

Choriocarcinomas can be divided into gestational and non-gestational forms. The former reportedly occurs in 1 in 20,000 to 40,000 pregnancies. On the other hand, the latter is a non-seminomatous germ cell tumor, and the most common primary sites of choriocarcinoma in men are the testes (36 %), followed by the mediastinum (14 %), and gastrointestinal tract (13 %) [1,3]. Of all mediastinal tumors, 10–20 % are germ cell tumors, with choriocarcinomas accounting for approximately 2 %. Most of these tumors co-occur with other germ cell tumors, and isolated choriocarcinomas, as in our case, are rarer [4]. The incidence of primary choriocarcinoma peaks between the ages of 20 and 29 years, with a male predominance [3,5]. The International Germ Cell Consensus Classification (IGCCC) for prognosis is generally used for the risk classification for seminomas and non-seminomas in men. According to the IGCCC, the mediastinum being the primary site is a poor prognostic factor for non-seminomas, with a 5-year survival rate of 48 % [6]. However, choriocarcinomas are associated with a poorer prognosis than other non-seminomas because of their highly angioinvasiveness nature, which makes them prone to hematogenous metastasis [7]. In a clinicopathological study of eight patients with primary mediastinal choriocarcinomas, all patients died within 1–2 months [8]. Moreover, most patients died before diagnosis, as in our case [4].

Making a definitive diagnosis using a small amount of biopsy tissue, such as that obtained via bronchial and CT-guided biopsy, is difficult. This may be because choriocarcinomas often co-occur with other germ cell tumors, and the choriocarcinoma component is difficult to isolate [5]. Additionally, because choriocarcinomas have a high proliferative capacity and propensity to bleed, the tumor lesions, including metastases, are prone to extensive hemorrhage and necrosis; this is another reason why definitive diagnosis is difficult [9]. Therefore, harvesting larger tumor tissues are required for a definitive diagnosis. In our case, we looked for appropriate sites for cervical or axillary lymph nodes biopsy, but no detectable enlarged lymph nodes on the body surface were found. Recently, a cryobiopsy has been used to harvest larger tissue samples. In rare tumors, such as lymphoma and seminoma, a cryobiopsy has been reported to be more sensitive to make a diagnosis than transbronchial needle aspiration [10]. This finding indicates that a large amount of tumor tissue is needed to make a diagnosis of a rare tumor. However, we didn't perform a cryobiopsy because of concerns that it might induce massive tumor hemorrhage. Consequently, it was difficult to make a definitive diagnosis before death in our case.

On the other hands, elevated serum β-hCG levels have been reported in 96.4 % of patients with choriocarcinomas; it is considered to have the same diagnostic value as pathological examination and can be a critical diagnostic and prognostic marker with high specificity and sensitivity [5,11]. Therefore, if the serum β-hCG level is elevated in urgent cases where a biopsy is attempted but the diagnosis is not confirmed, primary management with chemotherapy for choriocarcinoma without histopathologic confirmation is appropriate [12]. In our case, CT findings showed extensive low-density areas within the tumors, indicative of necrotic changes, which made definitive diagnosis difficult. Furthermore, the serum β-hCG was not examined, because we considered the primary site to be the right upper lobe with mediastinal lymph node enlargement instead of the mediastinum, and suspected lung cancer. Thus, chemotherapy for non-small cell lung cancer was considered, but unfortunately not administered.

On pathological examination, choriocarcinomas are biphasic tumors composed of cytotrophoblasts, characterized by medium-sized cells often with an eosinophilic cytoplasm, and syncytiotrophoblasts, characterized by condensed nuclei and an acidophilic cytoplasm [[13], [14], [15], [16]]. However, when, as in our case, there are no cytotrophoblasts in the small biopsy tissue samples and only syncytiotrophoblasts are identified, adenocarcinoma is more likely to be suspected than choriocarcinoma [14]. Furthermore, unlike large cell carcinomas or hCG-producing lung tumors, choriocarcinoma are characterized by gross findings of primary and metastatic tumors with hemorrhage and histological findings of biphasic cells: syncytiotrophoblasts and cytotrophoblasts [14]. Consequently, our case exhibited these findings, which led to a diagnosis of choriocarcinoma.

## 4 Conclusion

We encountered an older case diagnosed with primary mediastinal choriocarcinoma at autopsy. Because the primary tumor was considered to be a right upper lobe tumor with mediastinal lymph node metastasis, lung cancer was suspected. However, a definitive diagnosis could not be obtained due to a variety of circumstances, including the extensive necrotic changes within the tumor. In patients with a tumor extending across the lung and mediastinum and exhibiting pathologic findings of a poorly differentiated carcinoma, we should examine serum β-hCG to consider choriocarcinoma. Furthermore, in urgent cases, aggressive treatment can be considered based on elevated β-hCG levels alone.

## CRediT authorship contribution statement

**Takahiro Matsuyama:** Writing – original draft, Visualization, Resources, Project administration, Investigation, Conceptualization. **Koji Kubota:** Resources, Investigation. **Kentaro Tsuruzono:** Resources, Investigation. **Hiroko Uchida:** Resources, Investigation. **Tetsuro Hamasaki:** Resources, Investigation. **Keiko Mizuno:** Writing – review & editing, Resources, Investigation. **Hiromasa Inoue:** Writing – review & editing, Supervision.

## Declaration of generative AI and AI-assisted technologies in the writing process

The authors disclose that they have no use of AI and AI-assisted technologies in the writing process.

## Declaration of competing interest

No conflict.

## References

[1] Copeland L.J., trophoblastic neoplasma Gestational, Copeland L.J., Jarrell J.F. (2000). Textbook of Gynecology.

[2] Zhang F., Zhang W., Shi H. (2014). Primary choriocarcinoma of the posterior mediastinum in a male: a case report and review of the literature. Oncol. Lett..

[3] Jiang F., Xiang Y., Feng F.Z., Ren T., Cui Z.M., Wan X.R. (2014). Clinical analysis of 13 males with primary choriocarcinoma and review of the literature. OncoTargets Ther..

[4] Fraser R.S., disease Mediastinal, Fraser R.S., Muller N.L. (1999). edsDiagnosis of the Chest.

[5] Yokoi K., Tanaka N., Furukawa K. (2008). Male choriocarcinoma with metastasis to the jejunum: a case report and review of the literature. J. Nippon Med. Sch..

[6] The International Germ Cell Cancer Collaborative Group (1997). International germ cell consensus classification: a prognostic factor-based staging system for metastatic germ cell cancers. International Germ Cell Cancer Collaborative Group. J. Clin. Oncol..

[7] Cao X., Feng H., Liu S., Chen L. (2023). Analysis of clinical characteristics and prognosis of 68 patients with primary pulmonary choriocarcinoma. BMC Pulm. Med..

[8] Moran C.A., Suster S. (1997). Primary mediastinal choriocarcinomas: a clinicopathologic and immunohistochemical study of eight cases. Am. J. Surg. Pathol..

[9] Qiu Z., Wu Y., Wang Y., Hu C. (2019). Male primary mediastinal choriocarcinoma with diffuse metastases: a case report. Méd..

[10] Zhang J., Guo J.R., Huang Z.S., Fu W.L., Wu X.L., Wu N., Kuebler W.M., Herth F.J.F., Fan Y. (2021 Dec 9). Transbronchial mediastinal cryobiopsy in the diagnosis of mediastinal lesions: a randomised trial. Eur. Respir. J..

[11] Wang H., Chen X., Zhang R. (2023). Primary mediastinal choriocarcinoma in an 18-year-old male with pulmonary and brain metastasis: a case report. Clin. Res. J..

[12] Nichols C.R. (1991). Mediastinal germ cell tumors. Clinical features and biologic correlates. Chest.

[13] Berkowitz R.S., Goldstein D.P. (1996). Chorionic tumors. N. Engl. J. Med..

[14] Miyayama H., Baba T., Kawano K. (1999). An autopsy case of primary choriocarcinoma of the lung in a man. J. Jpn. Soc. Clin. Cytol..

[15] Di Crescenzo V., Laperuta P., Napolitano F., Carlomagno C., Garzi A., Vitale M. (2013). An unusual case of primary choriocarcinoma of the lung. BMC Surg..

[16] Fujiwara Y., Okamoto K., Ninomiya I. (2020). Surgically resected primary esophageal choriocarcinoma accompanied with Barrett's adenocarcinoma: a case report. Surg. Case Rep..

